# The HDAC Inhibitor Butyrate Reduces SOD1 Aggregation and Improves Motor Function in *C. elegans* and Cellular Models of ALS

**DOI:** 10.1155/ijcb/3022967

**Published:** 2026-08-27

**Authors:** Fiona C. Dresel, Martin Michaelis, Campbell W. Gourlay

**Affiliations:** ^1^ School of Natural Sciences, University of Kent, Canterbury, Kent, UK, kent.ac.uk

## Abstract

Amyotrophic lateral sclerosis (ALS) is a progressive neurodegenerative disease characterised by motor neuron loss and protein aggregation, commonly driven by mutations in superoxide dismutase 1 (Sod1). Recent evidence implicates gut microbiota–derived metabolites, such as butyrate, in modulating neurodegeneration, but the underlying mechanisms remain unclear. Here, we demonstrate that sodium butyrate (NaB), a histone deacetylase inhibitor and microbial metabolite, ameliorates ALS‐related phenotypes in *C. elegans* and mammalian cell models expressing mutant isoforms of Sod1 linked to ALS. NaB treatment prevented Sod1 aggregation and restored motor function and axonal integrity in transgenic worms overexpressing Sod1^G85R^. Mechanistically, NaB recapitulated the effects of the pan‐HDAC inhibitor trichostatin A, suggesting HDAC inhibition as key to reducing Sod1 aggregation and its downstream effects. Application of NaB or the HDAC inhibitor valproic acid also prevented aggregation of Sod1^A4V^, Sod1^G85R^ or Sod1^G37R^ in transfected human neuroblastoma cells. These findings support a conserved neuroprotective role for NaB and HDAC inhibitors via their antiaggregation activity. Our findings also verify *C. elegans* and neuroblastoma cell lines as excellent research tools to explore the mechanisms underlying the antiaggregation action of NaB and HDAC inhibitors, as well as their potential for future therapeutic development.

## 1. Introduction

The neurodegenerative disorder amyotrophic lateral sclerosis (ALS) leads to a progressive loss of motor neurons controlling voluntary muscles responsible for eating, speaking, walking and breathing, ultimately leading to respiratory paralysis [1]. ALS, also known as Lou Gehrig Disease, has a prevalence in excess of 32,000 annually in the United States [2]. There are two types of ALS which are pathologically indistinguishable from one another, familial amyotrophic lateral sclerosis (fALS) and sporadic amyotrophic lateral sclerosis (sALS). fALS is caused by heritable mutations in a range of genes, the most common being in *C9orf72*, *SOD1*, *TARDBP* and *FUS*, whereas sALS accounts for around 90% of ALS cases [3, 4]. The prevalence of Sod1 mutations linked to fALS is significant but varies with populations. For example, within European populations, Sod1 mutations were reported to account for 14,8% case of fALS, whereas in Asian populations this was 30% [5]. Mutations in Sod1 have been detected in 1%–2% of sALS cases [6].

The evolutionary conserved cytosolic enzyme superoxide dismutase 1 (Sod1) is an important antioxidant and redox regulatory enzyme whose main role is to convert potentially harmful superoxide anions to hydrogen peroxide. However, a range of studies have also linked Sod1 activity to the control of signalling process, gene expression control and enzyme stability, making it an important enzyme in cellular homeostasis and adaptability [7–11]. *SOD1* was the first gene to be linked to fALS [12]. Since its first discovery, more than 217 mutations that are linked to fALS have been identified. These mutations can be found in all five exons of the *SOD1* gene and comprise point mutations, truncations, deletions and substitutions. The most common fALS‐linked mutations in *SOD1* are D90A, A4V and G93A, with the A4V mutation being the most frequently found in the United States and D90A around the rest of the world [13]. The mutation of Sod1 appears to lead to changes in stability that lead to its aggregation [14–16]. In its misfolded state, Sod1 exposes hydrophobic regions that are normally buried within its stable structure, leading to self‐association into soluble oligomers and, eventually, larger insoluble aggregates. Mutations in SOD1 that are linked to ALS can also decrease the ability of apo‐Sod1 monomers to form dimers and increase the rate of unfolding at physiological temperature [17]. Although aggregation is a common occurrence, mutations are associated with different disease severity, for example, the Sod1^A4V^ mutation is linked to rapid decline [18], whereas Sod1^G37R^, Sod1^D90A^ or Sod1^G93A^ are often linked to less severe disease pathology [19–21]. In some cases, Sod1 aggregation can follow a seeding mechanism, where small amyloid‐like fibrils accelerate the accumulation of additional Sod1 species [22]. Sod1 aggregation is generally cytotoxic and contributes to ALS pathology via disruption to a number of processes including redox regulation, increased oxidative stress, excitotoxicity, disrupted axonal transport, mitochondrial dysfunction and neuroinflammation [23, 24].

While the aetiology and pathophysiology of ALS are well documented, it has recently been demonstrated that the gut microbiota may play a role in disease progression [25]. The gut microbiota functions to absorb nutrients and minerals, synthesise amino acids, enzymes and vitamins, and to create short‐chain fatty acids (SCFAs). As such, it has a profound influence on human health and has been linked to a wide range of disease pathologies [26]. Variation in gut microbiota composition and activity may result in different metabolite profiles with a direct impact upon cell signalling pathways, and downstream effects that may contribute to or impede host health [27, 28]. Studies have identified that the microbiome of ALS patients differs from healthy controls. Levels of *Bacteroidetes* and *Firmicutes* are reported as significantly higher in healthy controls when compared with ALS patients. Furthermore, species *Oscillibacter*, *Anaerostipes* and *Lachnospiraceae* were substantially decreased in ALS patients [29]. It has been reported that dysbiosis and decreased diversity of the microbiome compared with healthy controls is observed in ALS patients [30]. Such findings have led to attempts to treat ALS patients with dietary interventions that might redress the dysbiosis, such as via probiotic supplementation with *Streptococcus and Lactobacillus* species [31].

SCFAs are a major energy source for gut epithelial cells and contribute 5%–10% of the total energy required by the human body [32–34]. SCFAs, such as butyrate, help to maintain a low luminal pH and promote an increase of microbial biomass as well as playing a critical role in mucus production and the colonising of beneficial bacteria [35]. Numbers of butyrate‐producing bacteria, including *Roseburia intestinalis* and *Eubacterium rectale* have been reported to be significantly lower in ALS patients when compared with healthy controls [36, 37]. Furthermore, ALS patients display reduced activity of enzymes involved in butyrate metabolism [37]. It may be that decreased butyrate production plays a key role in ALS pathology. Evidence to support this has been generated in a mouse model, with NaB treatment leading to an improvement in motor function in the Sod1^G93A^ ALS model system [38]. It is believed that one mode of action of NaB is through HDAC inhibition [39–43]. Other HDAC inhibitors such as valproic acid (VPA) and trichostatin A (TSA) have been considered as potential ALS treatments [44] whereby VPA has been tested in clinical trials [45, 46] and TSA in more lab‐based studies [44, 47, 48].

Considerable efforts have been made to try and prevent Sod1 aggregation as a route to preventing fALS, leading to the development of a range of model systems, such as *S. cerevisiae* [49, 50], *C. elegans* [51–53] and cell‐based models [54, 55]. In this study, we aim to investigate the effect of NaB supplementation on Sod1 aggregation and motor function in transgenic Sod1^G85R^ *C. elegans* and in neuroblastoma overexpression models. We report that NaB supplementation could ameliorate motor and neuromuscular junction (NMJ) defects observed in transgenic Sod1^G85R^ *C. elegans* and could decrease visible Sod1 aggregates in *C. elegans* and neuroblastoma cells expressing fluorescent forms of the protein. Consistent with these effects being attributable to the known function of NaB as a histone deacetylase (HDAC) inhibitor, we could observe similar results with the HDAC inhibitors TSA and VPA. These findings demonstrate that *C. elegans* and cell‐based models may be employed to determine how NaB and other clinically used HDAC inhibitors function to limit Sod1 aggregate burden and toxicity, providing a platform for preclinical evaluation.

## 2. Materials and Methods

Within the manuscript, human Sod1 is referred to as SOD1 (protein) and *SOD1* (gene). Mutant proteins are denoted as SOD1^G85R^, SOD1^A4V^ and SOD1^G85R^. Transgenic expression in *C. elegans* is indicated as hSOD1 or hSOD1^G85R^.

### 2.1. *C. elegans* Strains and Maintenance

For *C. elegans* culture and maintenance, the uracil auxotroph *E. coli* strain OP50 was used. The N2 strain of *C. elegans* was used as a wild type strain control. The transgenic *C. elegans* strains expressing the hSOD1 protein and the hSOD1^G85R^ mutant protein in an integrated manner and both transgenes are C‐terminally fused to YFP. A pan‐neuronal promoter was used to express the proteins in the 302 neurons of a *C. elegans* hermaphrodite. Both strains have been well characterised in terms of Sod1 aggregation and were created by Professor Jiou Wang (John Hopkins Bloomberg School of Public Health). The strains are Psnb‐1::hSOD1‐YFP and Psnb‐1::hSOD1^G85R^‐YFP and were a kind gift from the lab of Professor Alan Morgan (University of Liverpool) [53]. The worms were maintained on standard NGM (0.3% w/v NaCl, 0.25% w/v bactopeptone, 1.7% w/v granulated agar, 2.5% v/v KH_2_PO_4_ (pH 6), 0.1% v/v MgSO_4_ (1 M), 0.1% v/v CaCl_2_ (1 M), 0.1% v/v cholesterol (5 mg/mL dissolved in ethanol)) containing 200 *μ*L *E. coli* OP50 at 20°C. Standard NGM agar plates were supplemented with the following additives after autoclaving: 0.5 mM aldicarb (Sigma‐Aldrich), 100 mM NaB (Sigma‐Aldrich) and 150 *μ*g/mL TSA (Cayman Chemical). For synchronisation, procedures plates containing plenty of eggs and gravid adult worms were washed with M9 buffer (3 g KH2PO4, 6 g Na2HPO4, 5 g NaCl, 1 mL 1 M MgSO4 and dH2O to 1 L) to obtain all worms in a centrifuge tube. Worm pellets were exposed to a 1:1 bleach 4 M NaOH for 5–6 min until the mothers have almost completely disintegrated. The bleach was immediately quenched by making the solution up to 15 mL with M9 buffer. To complete this wash step the tube was centrifuged at 3000 rpm for 1 min. The wash step was repeated twice and around 200 *μ*L of the resuspended M9/egg solution was pipetted onto fresh NGM OP50 plates or supplemented NGM OP50 plates.

### 2.2. Synaptic Transmission Assay Using Aldicarb

NMJ function can be studied in *C. elegans* using aldicarb which inhibits acetylcholinesterase, causing acetylcholine accumulation in the synaptic cleft. NGM plates containing 0.5 mM aldicarb were prepared 1 day before use and stored at 4°C. Thirty to forty L4 stage larvae of strains of interest were picked using a sterile platinum wire and transferred onto fresh NGM plates seeded with OP50 *E. coli* with or without supplements (100 mM NaB or 150 *μ*g/mL TSA) but not aldicarb. The worms were then incubated at 20°C for 24 h. Copper rings were then dipped into 70% ethanol and flamed for a few seconds before placing them onto the 0.5 mM aldicarb containing NGM plate. A total of 10 *μ*L of OP50 *E. coli* were pipetted into the centre of the ring to keep the worms in this area. Twenty Day 1 adults of each genotype were transferred to the centre of each ring. The worm transfer was staggered every 3 min so that all three phenotypes could be examined within an interval of 10 min. Worms were counted as paralysed and removed from the plate when they failed to move their head even if touch was applied. For each experiment, data from three independent biological replicates are included, with the sample sizes indicated in figure legends. A two‐way repeated measures ANOVA applying the Geisser–Greenhouse correction and using a Tukey′s post hoc multiple comparison test was used to measure statistical significance.

### 2.3. *C. elegans* Thrashing Assay

Using bleach, L4 worms were age synchronised and time points at Days 1, 2, 6 and 7 of adulthood were used to perform this experiment. At each time point, 15 worms were tested individually. A total of 200 *μ*L of M9 buffer were pipetted into each well of a 96‐well plate. One worm at a time was picked and transferred onto an unseeded plate to remove any OP50 attached to the worm. The worm was then placed into one of the wells and left to settle for 1 min before counting its body bends for 30 s. A thrash was characterised when the worm moved its head and tail towards each other. For each experiment, data from three independent biological replicates are included, with the sample sizes indicated in figure legends. A one‐way repeated measures ANOVA (RM‐ANOVA) applying the Geisser–Greenhouse correction and using a Tukey′s post hoc multiple comparison test was used to measure statistical significance.

### 2.4. Confocal Microscopy of *C. elegans*

*C. elegans* were grown on either standard NGM OP50 plates or NGM OP50 supplemented with 100 mM of NaB or 150 *μ*g/mL TSA. Worms were picked and immobilised using 0.2% tricaine combined with 0.02% levamisole in M9 buffer. The anaesthetized worms were then transferred onto an agarose pad on a glass slide. A coverslip was then placed on top of the worms leaving two small gaps along the sides to allow for gas exchange. The slides were viewed under a Zeiss LSM880/Elyra/Axio Observer.Z1 Confocal Microscope (Carl Zeiss Inc.) using the 488‐nm argon and the 561‐nm DP55 lasers and 60× objective. The image acquisition software used was ZENBlack (Carl Zeiss Inc.), and the image processing software was ZENBlue (Carl Zeiss Inc.). Z‐stacks were taken allowing for collection of multiple images across the entire thickness of the worm. Experiments were conducted in biological triplicate and 10 worms analysed per experiment. Image J was used to analyse the measurement of mean fluorescence intensity (MFI) in a standardised region of interest that covered the head ganglia and ventral cord. Control and treated samples were acquired using the same acquisition parameters. The background MFI was subtracted from the MFI of the region of interest to avoid contribution to the “true” signal of the area of interest.

### 2.5. Culture and Maintenance of SH‐SY5Y Neuroblastoma Cells

The human neuroblastoma cell line SH‐SY5Y was derived from ATCC (Manassas, United States) and maintained in Iscove′s Modified Dulbecco′s (IMDM; Fisher Scientific, United Kingdom) supplemented with 10% (v/v) foetal bovine serum (FBS; Sigma‐Aldrich, Germany), 100 IU/mL penicillin and 100 *μ*g/mL of streptomycin (Life Technologies, United Kingdom; cell culture medium), at 37°C in a humidified 5% CO_2_ incubator. 70%–80% confluent cultures were washed with phosphate buffered saline (PBS), detached with 0.05% w/v aqueous trypsin‐EDTA solution (Sigma‐Aldrich, Germany) and reseeded at an appropriate ratio into a new flask. To prevent genetic deviation, cell populations were passaged continuously for no longer than 5 months. Fresh stocks were made from a subpopulation of each cell line from the earliest passage possible.

### 2.6. Cell Viability Assay

Cell viability was measured by a (3‐(4,5‐dimethylthiazol‐2‐yl)‐2,5‐diphenyltetrazolium bromide) MTT assay modified after Mosmann [56] as previously described [57]. Here, the yellow tetrazole 3‐(4,5‐dimethylthiazol‐2‐yl)‐2,5‐diphenyltetrazolium bromide (MTT; Universal Biologicals United Kingdom) is metabolically reduced to purple formazan only in living cells [56]. Five thousand cells/well suspended in 200 *μ*L of cell culture medium were plated per well in 96‐well plates and incubated in the presence of the indicated drug concentrations. The 96‐well plates were then incubated at 37°C for 5 days or until the max wells had a confluency of 80%. Then, 25 *μ*L of MTT reagent (2 mg/mL MTT (w/v) in PBS) was added and cells were incubated for 4 h. Subsequently, the cells were lysed using 200 *μ*L of sodium dodecylsulfate (SDS, Fisher Scientific) buffer (20% (w/v) SDS in 50% (v/v) N, N‐dimethylformamide, pH adjusted to 4.7 at 37°C) and incubated overnight. Absorbance was read at 600 nm wavelength in a Victor X4 Multilabel Plate reader (PerkinElmerLife Sciences, United States).

### 2.7. Preparation of Total Cell Lysates and Immunoblotting

Cells were grown to a confluency of 70% in TF25 flasks. The cells were washed with ice‐cold PBS twice after the culture medium was removed and exposed to 100 *μ*L of lysis buffer (50 mM HEPES pH 7.4, 250 mM NaCl, 0.1% NP40, 1 mM DTT, 1 mM EDTA, 1 mM NAF, 10 mM *β* Glycerophosphate, 0.1 mM sodium orthovanadate and Complete protease inhibitor cocktail [Roche, Switzerland]). The cells were scraped into cold microcentrifuge tubes and incubated on ice for 30 min before centrifugation at 13,000 rpm for 10 min at 4°C to remove insoluble material. The lysate was then transferred to a sterile microcentrifugation tube and kept on ice or frozen on dry ice and stored at −80°C. Protein concentrations were determined by sodium salt bicinchoninic acid (BCA) method. Thirty *μ*g/well of proteins were separated on 4%–12% SDS‐polyacrylamide gels and transferred to PVDF membranes (Thermo Fisher). The PVDF membranes were placed in blocking solution (5% w/v dried skimmed milk [Oxoid] and PBS) for 45 min with rotation at room temperature followed by incubating in blocking solution containing the H4 primary antibody (BioRad AHP418) in a 1/800 dilution overnight at 4°C. The following day, membranes were washed with PBS/T (phosphate buffered saline and 0.2% Tween 20) before being exposed to the horseradish peroxidase conjugates antirabbit secondary antibody (Sigma 12‐348, 1/10000) for 45 min at room temperature and further washed 6× with PBS/T. The immunoreactivity was visualised using enhanced chemiluminescence (ECL) using a SynGene gel doc. To analyse the blots the GeneSys Software (Version 1.6.5.0) was used, and a Synoptics 6MP camera was used to capture the images. The programme Fiji (ImageJ) version (2.1.0/1.53c) was used to normalise to the control by calculating the integrated intensity of the protein band which was then divided by the integrated intensity of the loading control band of anti–alpha‐tubulin antimouse antibody (Sigma‐Aldrich).

### 2.8. Transfection of SH‐SY5Y Cells

We generated transfected SH‐SY5Y cells containing Sod1^mut^ using the Ionza nucleofector tool kit. The transfected cells were selected using G418 in cell culture medium. All transgenes are C‐terminally fused to GFP and a pAcGFP1‐N1 vector backbone was used. The Lonza Amaxa Cell Line Nucleofector Kit V and the amaxa nucleofector II transfection device were used to transfect the cell line SHSY5Y. Prenucleofection procedures were followed per manufacturer′s instructions for SH‐SY5Y cells: The media was changed twice a week, and cells were passaged as normal at 75%–80% confluency. Cells were seeded out at 2 × 10^5^ cells/cm^2^. Cells were again grown to 75%–80% confluency. Medium was then removed from the cultured cells and washed once with PBS. The cells were then trypsinised as usual for about 5 min at 37°C. The trypsin was inactivated with cell culture medium once the cells had detached. For one nucleofection sample, 1–2 × 10^6^ cells, 2 *μ*g plasmid DNA and 100 *μ*L Cell Line Nucleofector Solution V were needed. T75 cell flasks containing 400 *μ*g/mL G418 in cell culture medium were prepared and preincubated in a humidified 37°C/5% CO_2_ incubator. A total of 1–2 × 10^6^ cells were centrifuged at 90 × g for 10 min at room temperature. After removal of the supernatant, the pellet was resuspended in 100 *μ*L Nucleofector Solution. Combining 100 *μ*L of this cell suspension with 2 *μ*g plasmid DNA, the cell/DNA mix was transferred into certified cuvettes. The Nucleofector Program A‐023 was used for high viability. Moreover, 500 *μ*L of prewarmed medium was added immediately after transfection, and the whole sample was transferred either into the pre‐equilibrated cell flasks or chambered cover slips in the 37°C/5% CO_2_ incubator. Cells were analysed 24 h post transfection. The plasmids used for transfection were pF146 pSOD1WTAcGFP1, pF147 pSOD1^A4V^AcGFP1, pF149 pSOD1^G85R^AcGFP1, pF150 pSOD1^G93A^AcGFP1 and pF148 pSOD1^G37R^AcGFP1, which contain well‐characterised mutations in SOD1 that are linked to fALS. All plasmids were obtained from Addgene.

### 2.9. Widefield Fluorescence Microscopy

After transfection, cells transferred into a chambered cover slip with eight individual wells and high walls for cell culture (ibidi) were left untreated or 5 mM NaB or 1 mM VPA treatment was added. Cells were incubated for 24 h before being analysed using widefield microscopy. Images were captured using an Olympus IX81 fluorescence microscope using either GFP filter set (395/509 excitation/emission) or using DIC at 100× magnification using an Andor Xyla 4.2 CMOS digital camera.

## 3. Results

### 3.1. The Impact of NaB Supplementation on SOD1‐Mediated Motor Defects in an ALS *C. elegans* Model

It has been reported that ALS patients lack butyrate producing bacteria [36] and that NaB supplementation could improve motor function in a mouse model of ALS [58]. In order to develop more tractable models to elucidate the effects of NaB, we investigated the effects of its supplementation in a *C. elegans* model of ALS. The concentration of NaB used (100 mM) has been previously shown as effective in previous *C. elegans* proteostasis studies [59]. We conducted a thrashing assay in untreated and NaB‐treated transgenic animals to provide the first insights into its effect on hSOD1 or hSOD1^G85R^‐induced motor defects in a *C. elegans* model of ALS.

**Figure 1:**
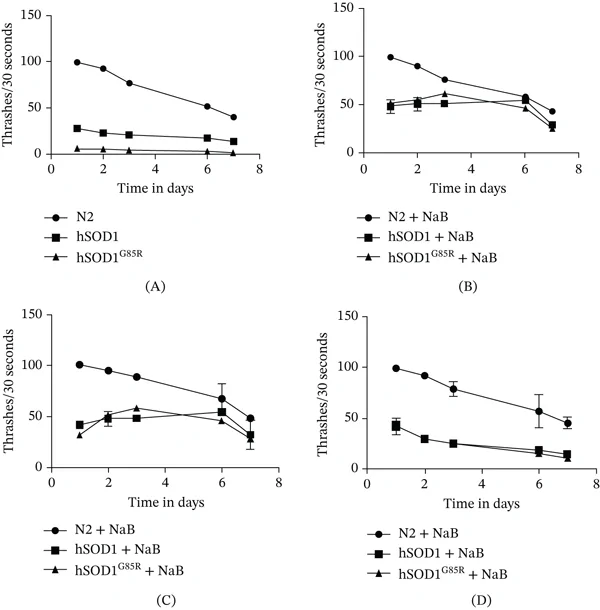
Thrashing assay showing the effect of 100 mM NaB and NaB withdrawal on transgenic ALS *C. elegans* strains. The body bends of 15 worms in liquid medium (M9 media) were recorded at 30‐s intervals for each strain on Days 1, 2, 3, 6 and 7. Age synchronisation was performed prior to the experiment by picking 100 L4 larvae per strain and per plate. Transgenic strains were compared with the standard N2 strain. The data represented display an average of the three biological repeats (*n* = 15 per repeat). The error bars indicate the standard deviation. A one‐way repeated measures ANOVA (RM‐ANOVA) applying the Geisser–Greenhouse correction and using a Tukey′s post hoc multiple comparison test was used to measure statistical significance. (A) Untreated animals. (B) Constant NaB exposure since hatching. (C) NaB exposure since early adulthood. (D) NaB withdrawal after one generation.

As reported in previous studies [51, 53], the overexpression of either hSOD1 or hSOD1^G85R^ led to decreased thrashing frequencies over a 7‐day time‐course when compared with the wild type N2 worm (Figure 1A). The hSOD1^G85R^ overexpression animals had the most severe thrashing impairment compared with both strains and would curl up or only move their head when gently prodded instead of moving freely in a sinusoidal pattern seen in the wild type N2 and h:Sod1‐overexpression worms [51, 53].

To determine the effects of NaB activity three separate thrashing assays experiments were devised (Figure 1B–D). In the first instance, worms were grown and eggs transferred onto either NGM or NGM NaB‐supplemented plates. This meant that the synchronised embryos transferred onto NaB plates were exposed to NaB from hatching and throughout development (Figure 1B). NaB supplementation had no significant effect on the thrashing activity of the N2 wild type strain (Figure 1B). The thrashing defect phenotype observed in both hSOD1 or hSOD1^G85R^ overexpression animals could be rescued by around 30% and 50%, respectively, when grown on 100 mM NaB‐supplemented plates. A significant difference in thrashing behaviour was still observed when hSOD1 and hSOD1^G85R^ NaB‐treated animals were compared with the wild type strain, suggesting a partial rescue of motor activity (Figure 1B). In addition, the mobility issues shown by hSOD1 or hSOD1^G85R^ worms were also markedly improved when supplemented with NaB (Movie S1).

Following this, we cultivated the animals on standard NGM OP50 plates from hatching until they reached their L4 larvae stage. The animals were then transferred onto NaB‐supplemented plates prior to assessing thrashing activity from Day 1 of adulthood (Figure 1C). In this experiment, the transgenic animals were only exposed to NaB from early adulthood. We could identify about a 50% rescue in thrashing in hSOD1^G85R^ overexpression animals upon NaB exposure, albeit with a slight delay compared with the first experiment (Figure 1A–C) where animals were exposed to NaB since hatching. However, on Days 6 and 7 the transgenic mutant NaB‐treated hSOD1^G85R^ worms behaved similarly to the untreated N2 control (Figure 1A,C). In the third thrashing experiment (Figure 1D), transgenic L4 larvae were cultivated on NaB‐supplemented plates for one generation and then transferred back onto untreated NGM plates. We found that sudden NaB withdrawal reversed the rescue effects in both hSOD1 and hSOD1^G85R^ overexpression animals. However, interestingly the hSOD1^G85R^ animals maintained a slight increase in thrashing frequency when compared with untreated hSOD1^G85R^ animals (Figure 1D).

### 3.2. Investigating the Impact of NaB and the HDAC Inhibitor TSA on SOD1‐Induced NMJ Dysfunction

NMJ dysfunction is a common symptom in the early stages of ALS [60]. We therefore sought to examine NMJ function in a *C. elegans* model of ALS and the effects of NaB upon this. As NaB is a known HDAC inhibitor, we examined whether the well‐characterised HDAC inhibitor TSA had similar effects. The TSA concentration used (150 *μ*g/mL) was selected as it has been used successfully in a *C. elegans* model of Huntington′s disease, in which its supplementation showed a clear proteostatic effect [61]. Locomotion in *C. elegans* is exerted through excitatory motor neurons releasing acetylcholine and inhibitory motor neurons, which release GABA y‐aminobutyric acid [62, 63]. NMJ function can be studied in *C. elegans* using aldicarb which inhibits acetylcholinesterase, causing acetylcholine accumulation in the synaptic cleft. Inhibiting acetylcholinesterase by aldicarb application causes continuous muscle contraction and dysfunction of the NMJ can be determined by either aldicarb resistance or sensitivity. Typically, low synaptic transmission correlates to increased aldicarb resistance, whereas increased synaptic transmission may be characterised by increased aldicarb sensitivity or faster paralysis [64]. It should be noted that aldicarb does not distinguish presynaptic versus postsynaptic defects.

**Figure 2:**
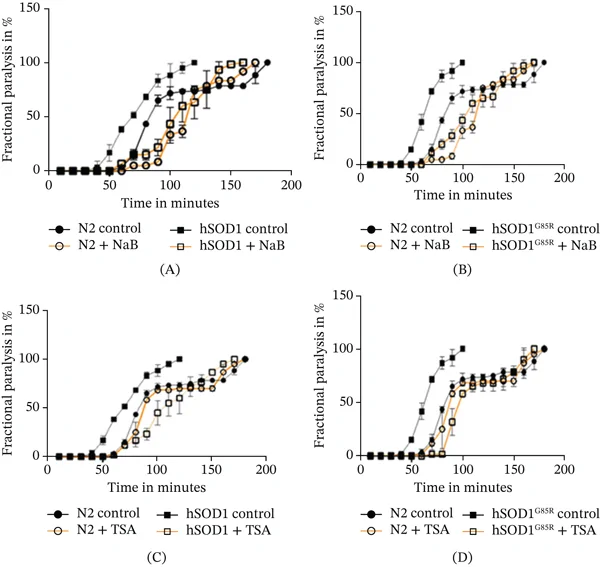
Aldicarb‐response curves of wild type N2 and transgenic animals overexpressing hSOD1 or hSOD11^G85R^ after 100 mM NaB or 150 *μ*g/mL trichostatin A (TSA) treatment. The control worms were grown on standard NGM OP50 plates and synchronised with the NaB or TSA plates. Age synchronisation was also performed prior to the experiment by picking 100 L4 larvae per strain and per plate. The experiment was carried out in triplicates, and the data represented display an average of the three biological repeats (*n* = 15 per repeat). The error bars indicate the standard deviation. A two‐way repeated measures ANOVA applying the Geisser–Greenhouse correction and using a Tukey′s post hoc multiple comparison test was used to measure statistical significance. (A–B) Aldicarb‐response curves after NaB exposure for transgenic hSOD1 and hSOD11^G85R^ animals. (C–D) Aldicarb‐response curves after TSA treatment for transgenic hSOD1 and hSOD11^G85R^ animals.

We observed that 100% of wild type worms achieved paralysis within 3 h when exposed to aldicarb (Figure 2A–D). The pattern of paralysis adopted a sigmoidal curve, suggesting that worms within a population may react differently to aldicarb exposure. Interestingly, although all animals were paralysed within 3 h, the treatment of wild type with NaB led to a reduction in aldicarb sensitivity within part of the population (Figure 2A–D). We could confirm that both transgenic ALS strains overexpressing either hSOD1 or hSOD1^G85R^ showed NMJ dysfunction when compared with the wild type (Figure 2A,B). Both hSOD1 and hSOD1^G85R^ transgenic strains were more aldicarb sensitive, achieving paralysis in 100% of the population within 120 and 100 min, respectively (Figure 2A,B).

Treatment of hSOD1 or hSOD1^G85R^ animals with NaB led to a wild type aldicarb resistance profile (Figure 2A,B) suggesting a rescue of the observed NMJ defect. Treatment of wild type *C. elegans* with 150 *μ*g/mL TSA led to no difference in aldicarb sensitivity (Figure 2C,D). However, as observed with NaB, TSA supplementation also led to a rescue in NMJ function in transgenic animals overexpressing either human hSOD1 or hSOD1^G85R^ that mirrored the wild type aldicarb response (Figure 2C,D).

### 3.3. The Effect of NaB and TSA Exposure on YFP‐hSOD1 and hSOD1^G85R^ Aggregate Presence in *C. elegans*

The effects of Sod1 stability and aggregation on cellular dysfunction and in the promotion of ALS pathology are well documented. As we had observed a significant improvement in motility and NMJ function, we sought to investigate whether NaB and TSA exposure had an effect on hSOD1 and hSOD1^G85R^ aggregation. We therefore carried out confocal microscopy to measure fluorescence intensity of YFP‐labelled hSOD1 or hSOD1^G85R^ animals in transgenic animals.

**Figure 3:**
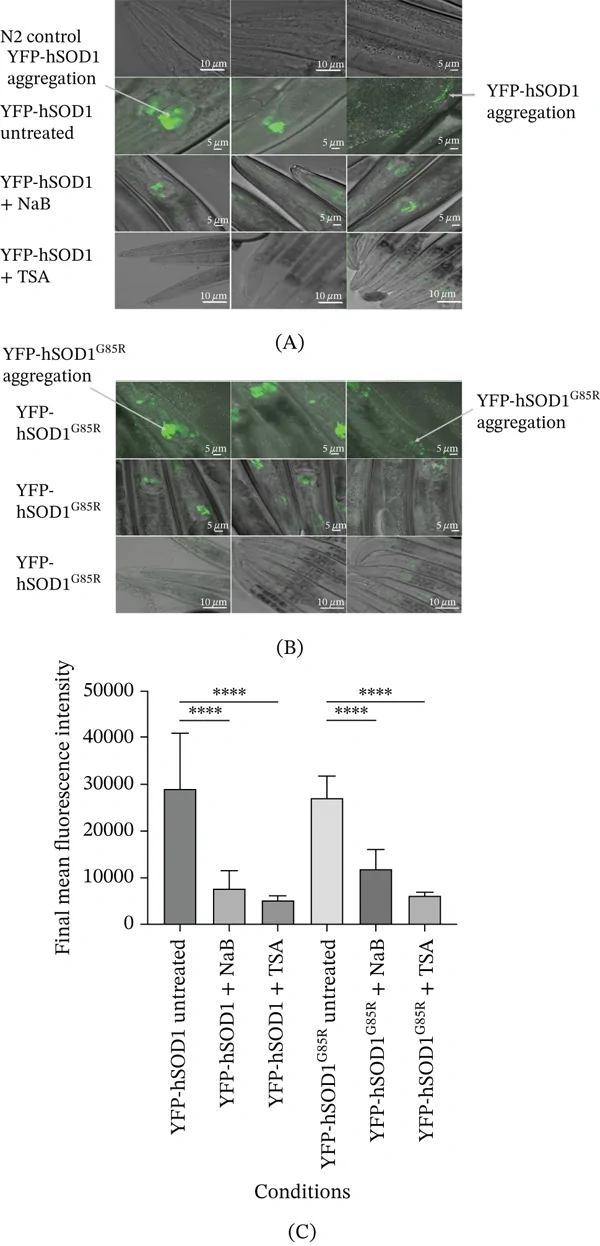
Effects of NaB and TSA on SOD1 aggregation in *C. elegans*. Confocal images and MFI of transgenic hSOD1 and hSOD11^G85R^. *C. elegans* grown with and without supplementation of 100 mM NaB or 150 *μ*g/mL trichostatin A (TSA). (A–B) Confocal images of untreated and NaB or TSA‐treated animals. (C) Mean fluorescence intensity (MFI) of YFP‐hSOD1 and YFP‐hSOD1^G85R^ expression. Each graph represents an average of three biological repeats (*n* = 10). The error bars display the standard deviation. A one‐way ANOVA using a Tukey multiple comparison test was used to determine statistical significance. (∗) flags levels of significance compared with the untreated strains.

The overexpression of YFP‐hSOD1 or YFP‐hSOD1^G85R^ in *C. elegans* led to the presence of visible fluorescent aggregations, which have been shown to be composed of aggregated SOD1 using biochemical approaches in previous studies [51, 53]. Clear fluorescent aggregations of YFP‐hSOD1 or YFP‐hSOD1^G85R^ were visible in the head ganglia and ventral cord (Figure 3A–C). Upon 24 h of exposure with NaB, we observed a significant and reproducible reduction in YFP‐hSOD1 and YFP‐hSOD1^G85R^ fluorescent aggregations within animals (Figure 3A–C). A significant and reproducible reduction in observable YFP tagged YFP‐hSOD1 or YFP‐hSOD1^G85R^ fluorescent aggregations was also observed upon exposure to TSA for 24 h (Figure 3A–C).

### 3.4. The Effect of NaB and the HDAC Inhibitor VPA on YFP‐SOD1 Aggregation in SH‐SY5Y Cells

Our results demonstrate that both NaB and TSA treatment led to a significant reduction in the presence of YFP‐hSOD1 and YFP‐hSOD1^G85R^ YFP fluorescent aggregations and to the improvement of associated motor defects in *C. elegans*. We wished to determine if these findings could be extended to a second ALS model system and so we tested the effects of NaB and the HDAC inhibitor VPA on the neuroblastoma cell line SH‐SY5Y transiently transfected to overexpress GFP‐tagged SOD1, SOD1^A4V^, SOD1^G85R^ or SOD1^G37R^. We first performed MTT assays to identify nontoxic working concentrations for both NaB and VPA in SH‐SY5Y cells, using NaB and VPA concentrations ranging from 0.07–5 mM (Figure S1A). A total of 1 mM was selected for VPA and 5 mM for NaB as working concentrations that showed minimal effects on viability. Our experiments were designed to test the potential for NaB and VPA to affect protein aggregation and not as a means to informing their potential for therapeutic use.

We hypothesised that Sod1 aggregation inhibition may occur as a result of HDAC inhibition. We therefore examined whether the selected concentrations of 1 and 5 mM for VPA NaB, respectively, demonstrated HDAC inhibition in a western blot analysis to detect H4 acetylation (Figures S1B and 4A). We could see that both 5 mM NaB and 1 mM VPA treatment significantly increased H4 acetylation in SH‐SY5Y cells compared with the untreated control (Figures S1B and 4A).

**Figure 4:**
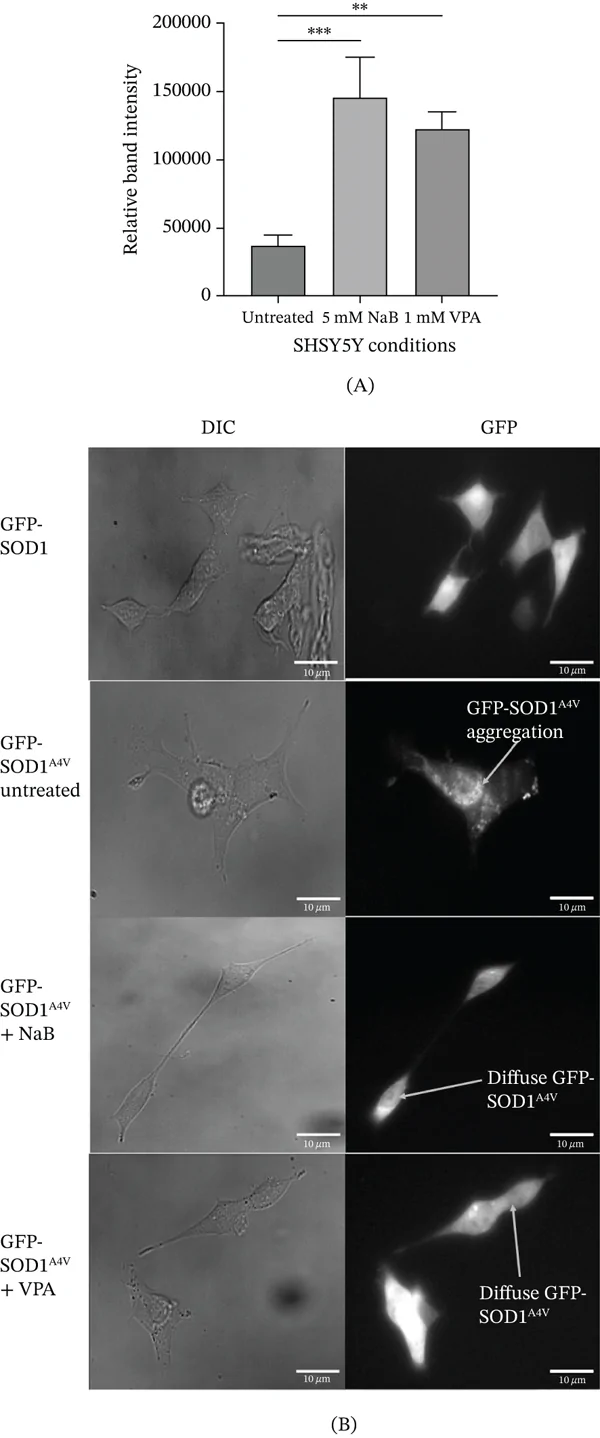
Effects of NaB and VPA on GFP tagged SOD1 and SOD1^A4V^ aggregation in SH‐SY5Y cells. (A) A bar chart representing the change in H4 acetylation relative to *α*‐tubulin loading control in SH‐SY5Y following exposure to either 1 mM VPA or 5 mM NaB for 24 h. The data represent an average of three biological repeats. The error bars display the standard deviation. A one‐way ANOVA using a Tukey multiple comparison test was used to determine statistical significance. ∗∗*p* < 0.005, ∗∗∗*p* < 0.0005. (B) Widefield DIC and fluorescence images of GFP‐tagged SOD1 and SOD1^A4V^‐transfected SH‐SY5Y cells. Cells were incubated in standard IMDM media supplemented with G418 to select for successfully transfected cells. The cells were then incubated with either 5 mM NaB or 1 mM VPA for 24 h. This experiment was repeated in biological triplicate and representative images are shown. The slides were viewed under an Olympus 1X81 inverted microscope and 60× objective.

**Figure 5:**
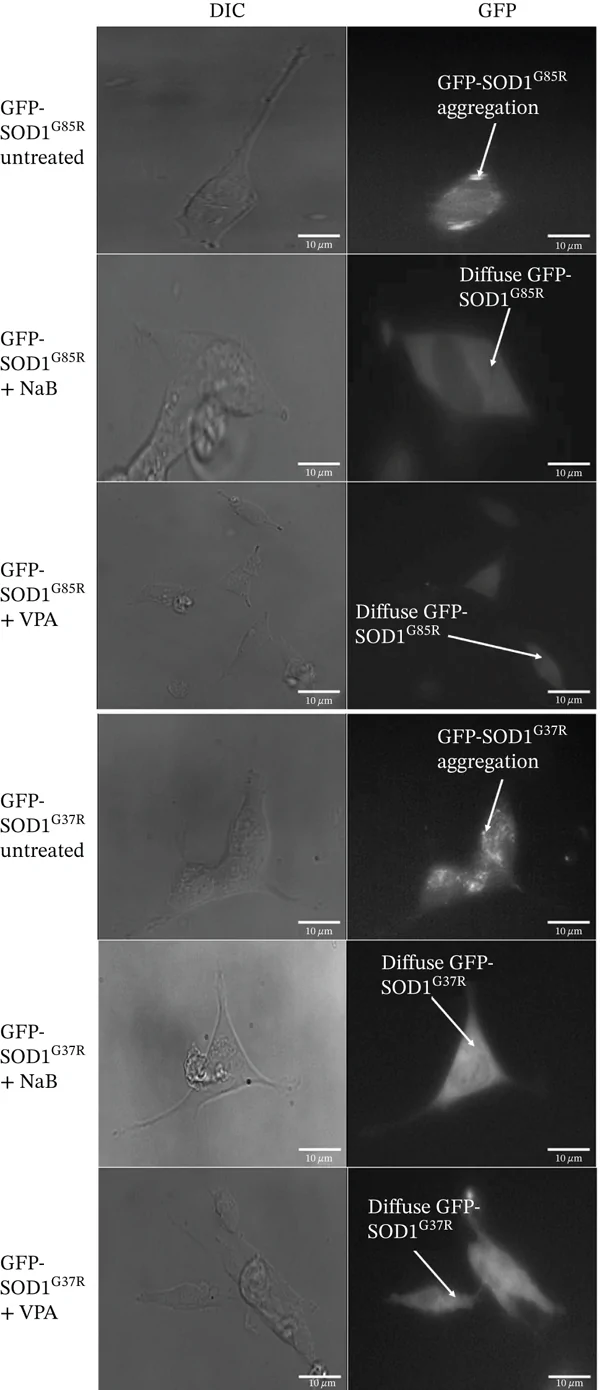
Effects of NaB and VPA on GFP tagged SOD1^G85R^ and SOD1^G37R^ aggregation in SH‐SY5Y cells. Cells were incubated in standard IMDM media supplemented with G418 to select for successfully transfected cells. The cells were then incubated with either 5 mM NaB or 1 mM VPA for 24 h. This experiment was repeated in biological triplicate and representative images are shown. The slides were viewed under an Olympus 1X81 inverted microscope and 60× objective.

We then conducted widefield fluorescence microscopy after transfecting GFP‐tagged SOD1, SOD1^A4V^, SOD1^G85R^ or SOD1^G37R^ expression plasmids into SH‐SY5Y cells and incubating for 24 h with or without the presence of either 5 mM NaB or 1 mM VPA. As expected, untreated and 5 mM NaB‐treated SOD1 expression in SH‐SY5Y cells displayed a diffuse cytoplasmic localisation with some clear signal in the nucleus (Figure 4B). The overexpression of SOD1^A4V^ led to clear fluorescent aggregations which were not present when incubated with either NaB or VPA (Figure 4B). Similar results were obtained in cells transfected with SOD1^G85R^ or SOD1^G37R^, with a clear reduction in fluorescent aggregations when cells were incubated with NaB or VPA (Figure 5).

## 4. Discussion

In the present study, we examined the effects of NaB on SOD1‐associated toxicity across both *C. elegans* and mammalian cell models. NaB treatment consistently reduced the abundance of visible mutant SOD1 fluorescent aggregates. In the *C. elegans* model, this reduction was accompanied by improved motor performance and improved neuromuscular function. The observation that similar antiaggregation effects were observed with the HDAC inhibitors TSA and VPA further suggests that modulation of protein homeostasis pathways may underlie the observed behavioural and cellular phenotypes. Overall, our experimental evidence adds to existing evidence that supports a role for the microbiome in ALS disease onset and progression [65–70]. For example, SOD1G93A expressing mice display changes in the gut microbiome at early stages of disease progression, prior to muscle atrophy and motor dysfunction [71]. In addition, reduced levels of butyrate‐producing microbes are reported in ALS patients [29, 72] alongside a decrease in butyrate metabolism [37]. Butyrate is one of the main SCFA generated through fermentation of dietary carbohydrates and fibres in the proximal colon, providing 60%–70% of the energy requirements of their epithelial cells [73–76]. SCFAs are also crucial for the protection and maintenance of the gut health [77], SCFAs are also crucial for the protection and maintenance of the gut health [77], which raises the possibility that dysbiosis and the resultant SCFA reduction may contribute to ALS pathology. However, it is equally plausible that systemic metabolic alterations, gastrointestinal dysfunction, altered diet or disease‐associated physiological changes that occur during disease progression also modify gut microbial communities, presenting a complex and bidirectional relationship. It is therefore likely that interventions designed to correct ALS associated dysbiosis will require a precise and personalised approach.

In the present study, we sought to investigate the impact of NaB supplementation on Sod1‐mediated aggregation and toxicity in animal and cell culture models of ALS. We note that the concentrations of NaB used in our study are higher than typical systemic and physiological gut levels. For example, human colonic butyrate is reported to be ~10–20 mM [78]. However, NaB has been administered at levels above 100 mM in human trials and been shown offer benefits to patient gut health [79]. Our data therefore establish a proof‐of‐principle for HDAC inhibition and aggregate reduction by NaB may be possible at levels that are achievable and well tolerated in the gut. The administration of NaB, or the widely used HDAC inhibitors TSA or VPA, led to reduced fluorescent Sod1 aggregations in both *C. elegans* and SH‐SY5Y ALS models, consistent with a proteostatic effect. Although SH‐SY5Y cells are widely used for neurodegeneration studies and provide a useful platform for discovery, we recognise that future studies using differentiated SH‐SY5Y cells or iPSC‐derived motor neurons will be required. We also observed a profound rescue of paralysis and NMJ dysfunction in *C. elegans* overexpressing SOD1G85R when treated with NaB or TSA. Withdrawal of NaB following one generation of exposure largely reversed the beneficial effects on locomotion, indicating that its beneficial effects are not a result of a stable correction of the underlying pathogenic state. Future studies using this *C. elegans* model may elucidate the underlying mechanism of NaB and TSA action on aggregate dynamics. We show that NaB, TSA and VPA application can reduce the abundance of visible SOD1‐associated fluorescent aggregates in two independent model systems. Although HDAC inhibition represents one plausible explanation for the observed effects, the present study did not directly investigate the downstream molecular mechanisms. Consequently, our data demonstrate an association between HDAC inhibitor exposure and reduced aggregate burden, but do not provide direct evidence for cause and effect, raising the possibility that HDAC independent mechanisms may be involved. In addition to histone acetylation, butyrate′s neuroprotective effects may involve other proteostasis pathways. For example, NaB has been shown to have beneficial effects on *C. elegans* proteostasis that require the SKN‐1/Nrf2 and DAF‐16/FOXO transcription factors [59]. Furthermore, NaB has been shown to activate autophagy in models of proteinopathy, promoting degradation of toxic aggregates [80]. Previous research has shown that NaB exposure can provide beneficial effects in mouse models of ALS. For example, the administration of NaB could restore intestinal function and increase life span in SOD1G93A expressing mice [58]. Another SOD1G93A ALS mouse study focusing on NF‐kappaB and bcl‐2 demonstrated that sodium phenylbutyrate increased life span and shapes antiapoptotic gene expression through HDAC inhibition [81]. Interestingly, a study using *Drosophila melanogaster* showed that TSA and NaB both influence autophagy and chaperones assisting in correctly folding newly synthesised proteins [82]. In addition, a study using motor neuron‐like NSC34 and HEK293 cell lines showed that HDAC6 knockdown led to an increase mutant SOD1 aggregation [83]. These data suggest that the correct use of NaB or suitable HDAC inhibitors may prove useful in reducing aggregation of Sod1. However, more clarity on the mode of action by which NaB and HDAC inhibition prevents aggregation is required to tailor use and improve ALS outcomes. This is highlighted by the fact that of the limited clinical trials have been conducted to assess NaB application in ALS patients there have been variable outcomes. A trial of phenyl butyrate NaPB showed little to no improvement in ALS disease progression [84], whereas a combination study using NaPB together with tauroursodeoxycholic acid (TUDCA) showed significant improvements in ALS patients but whether this was as a result of NaB alone could not be differentiated [85, 86].

In conclusion, we provide further evidence that NaB prevents the aggregation and downstream detrimental effects of several mutant isoforms of Sod1 that are linked to ALS onset. Further study using the model systems described here may help to model provide a much needed mechanism to understand how NaB and HDAC inhibition may be employed to target Sod1 aggregation. Future work should determine whether altered chaperone expression, enhanced autophagic clearance, modulation of proteostasis networks or other downstream consequences of HDAC inhibition contribute to the reduction in aggregate burden observed here. The models utilised in this study provide a platform for future studies that may identify microbial species that are important in producing antiaggregation metabolites with a view to tackling the dysbiosis that is observed in ALS patients and linked to poor outcome.

## Author Contributions

F.C.D. conceived and conducted all experimental procedures and analyses and wrote the manuscript. C.W.G. and M.M. assisted in conception of experimental design, supervision and manuscript editing.

## Funding

This study was supported by Biotechnology and Biological Sciences Research Council, 10.13039/501100000268.

## Conflicts of Interest

The authors declare no conflicts of interest.

## Supporting Information

Additional supporting information can be found online in the Supporting Information section.

## Supporting information


**Supporting Information 1** Figure S1: Dose‐response curve of NaB and VPA and western blot analysis of SH‐SY5Y after 5 mM NaB or 1 mM VPA treatment. (A) Dose‐response curve of NaB and VPA in SH‐SY5Y testing concentrations ranging from 0.07 to 5 mM in a MTT assay, data represent fold change in MTT absorbance relative to untreated control. (B) Western blot showing the detection of H4 in SH‐SY5Y cells after 5 mM NaB or 1 mM VPA treatment after 24 h. For the western blot analysis, cells were cultured in IMDM and were sampled after 24 h of NaB or VPA treatment. Uniform protein loading was ensured by probing levels of *α*‐tubulin after probing for H4. The dose‐response curves represent an average of three biological repeats. The error bars display the standard deviation. A one‐way ANOVA using a Tukey multiple comparison test was used to determine statistical significance. (∗) flags levels of significance compared with the untreated cells.

**Supporting Information 2** Movie S1: The movies display representative examples of experiments to test the effects of NaB or TSA supplementation on thrashing and motility in hSOD1 and hSOD11^G85R^ expressing in *C. elegans*. The movies show that the thrashing and motility defect generated by expression of hSOD11^G85R^ is clearly improved by addition of NaB or TSA at the stated doses; this effect is quantified following analysis of multiple animals in Figure 1.

## Data Availability

The data that support the findings of this study are openly available in Kent Academic Repository at https://kar.kent.ac.uk/.
